# Assessing the Accuracy of Sales Forecasts Submitted by Pharmaceutical Companies Applying for Reimbursement in Austria

**DOI:** 10.3389/fphar.2021.726758

**Published:** 2021-08-13

**Authors:** Michael Kossmeier, Madeleine Themanns, Lena Hatapoglu, Bernhard Kogler, Simon Keuerleber, Jutta Lichtenecker, Robert Sauermann, Anna Bucsics, Michael Freissmuth, Eva Zebedin-Brandl

**Affiliations:** ^1^ Federation of Social Insurances, Vienna, Austria; ^2^ MoCA (Mechanism of Coordinated Access to Orphan Medicinal Products), Brussels, Belgium; ^3^ Institute of Pharmacology, Centre of Physiology and Pharmacology, Medical University of Vienna, Vienna, Austria

**Keywords:** reimbursement of pharmaceuticals, reimbursement submission dossier, sales forecasts, forecast error, accuracy ratio, degree of innovation, therapeutic value, market dynamics

## Abstract

**Objectives:** Reimbursement decisions on new medicines require an assessment of their value. In Austria, when applying for reimbursement of new medicines, pharmaceutical companies are also obliged to submit forecasts of future sales. We systematically examined the accuracy of these pharmaceutical sales forecasts and hence the usefulness of these forecasts for reimbursement evaluations. **Methods:** We retrospectively analyzed reimbursement applications of 102 new drugs submitted between 2005 and 2014, which were accepted for reimbursement outside of hospitals, and for which actual reimbursed sales were available for at least 3 years. The main outcome variable was the accuracy ratio, defined as the ratio of forecasted sales submitted by pharmaceutical companies when applying for reimbursement to actual sales from reimbursement data. **Results:** The median accuracy ratio [95% confidence interval] was 1.33 [1.03; 1.74, range 0.15–37.5], corresponding to a median overestimation of actual sales by 33%. Forecasts of actual sales for 55.9% of all examined products either overestimated actual sales by more than 100% or underestimated them by more than 50%. The accuracy of sales forecasts did not show systematic change over the analyzed decade nor was it discernibly influenced by reimbursement status (restricted or unrestricted), the degree of therapeutic benefit, or the therapeutic area of the pharmaceutical product. Sales forecasts of drugs with a higher degree of innovation and those within a dynamic market tended to be slightly more accurate. **Conclusions:** The majority of sales forecasts provided by applicants for reimbursement evaluations in Austria were highly inaccurate and were on average too optimistic. This is in line with published results for other jurisdictions and highlights the need for caution when using such forecasts for reimbursement procedures.

## Introduction

To contain healthcare costs, European national payers endeavour to control pharmaceutical expenditures by using their resources efficiently (Godman et al., 2015; Nolte and Corbett, 2015; Vogler et al., 2017; Godman et al., 2018). Hence, payers must know whether new drugs have a therapeutic benefit compared to available alternatives. This is the basis for deciding if and under which conditions these new drugs are reimbursed. Health technology assessment of new medicines can be challenging; cancer drugs, for example, have been criticized for having very high prices despite uncertain value (Cohen, 2017). In addition, national payers need to know the likely uptake and respective costs of these new drugs. Budget impact models (BIMs) can support coverage decisions, but their role in health-care decision making varies across different jurisdictions (Cohen et al., 2008; Mauskopf et al., 2013; Faleiros et al., 2016; Flume et al., 2018; Foroutan et al., 2018; Ghabri and Mauskopf, 2018). Previous analyses raise concerns on the methodological quality and the accuracy of BI predictions and pharmaceutical forecasts (Cha et al., 2013; Broder et al., 2018). However, accurate forecasts and BI predictions are central for decision makers to adequately allocate resources and adapt budgets on the sectoral and on a national level. For example, the Swedish Early Awareness and Alert System identifies new medicines with substantial economic impact, in order to inform decision makers accurately about impending new medicines prior to their launch (Eriksson et al., 2017; Linner et al., 2020).

The Austrian Federation of Social Insurances (Dachverband der österreichischen Sozialversicherungen, DVSV) is a self-governing body, which has, inter alia, the legal responsibility of publishing the EKO (Erstattungskodex, “Code of Reimbursement”, (Allgemeines Sozialversicherungsgesetz (General Social Insurance Law), 2021). The EKO is a positive list of medicines reimbursed by the Austrian Social Insurances for outpatient care (Bucsics et al., 2016).

Market authorization holders are required to submit forecasts of expected sales when applying for listing in the EKO. Due to the very high grade of coverage, sales of innovative products reimbursed by Social Insurances essentially reflect almost all sales in the out-patient sector in Austria.

In the present study, we retrospectively compared sales forecasts submitted by pharmaceutical companies over a period of 10 years to actual reimbursement data in a large administrative database to assess the accuracy of the submitted forecasts.

## Methods

### Setting

This study was conducted in Austria, which has a universal “Bismarck”-type healthcare system covering more than 98% of the population, and this coverage includes appropriate and necessary pharmaceuticals. In practice, patients acquire prescribed medicines at a pharmacy or from a dispensing doctor, after paying a fixed prescription fee (unless they are exempt, or the medicine’s price is lower than the prescription fee). The dispenser submits monthly bills to Austrian Social Insurances, including information about the dispensed medicines.

The procedure for publishing the EKO is described in the VO-EKO (Verfahrensordnung zur Herausgabe des Erstattungskodex, (Verfahrensordnung zur Herausgabe des Erstattungskodex: Federation of Austrian Social Insurances, 2004). Listing of a new medicine in the EKO is preceded by a submission by the applicant (typically the marketing authorization holder), followed by a pharmacological, medical-therapeutical and health economic evaluation of the drug (which is based on the submitted data and documents), followed by price negotiations, a critical appraisal and a recommendation made by the Drug Evaluation Committee (representatives of stakeholders and academics). Based on this recommendation, a positive or negative decision on whether to list the drug is made by the DVSV. As the applicant applies for inclusion in the EKO, the outcome of the evaluations is either to accept the submission and reimburse the medicine, with or without restrictions, at an agreed price, or to reject the application. Products with unrestricted reimbursement are placed in the so-called “Green Box”; products which are in general more innovative and more expensive are reimbursed with restrictions and can be placed in the so-called “Yellow Box” for reimbursement under defined conditions.

Applicants are required to fill in application forms/data sheets electronically and to submit these online, together with appropriate documentation. The application includes a categorization of the degree of innovation of the medicine that is being submitted and of its added therapeutic benefit. In addition, the applicant is required to submit a forecast of the expected sales corresponding to the number of patients with the disease and the potential number of patients expected to be treated with the specified product. The sales forecast allows calculating the budget impact of the medicine submitted for reimbursement (Verfahrensordnung zur Herausgabe des Erstattungskodex: Federation of Austrian Social Insurances, 2004).

### Data Acquisition Procedure, Sources of Data, and Data Handling

A proprietary document management system (DOXIS™) is used during the application process for inclusion into the EKO. This secure and confidential electronic system is used to manage and store all relevant documents and information for applicants and evaluators of a given application procedure. Accordingly, it allows for a systematic retrospective analysis of procedures for publishing the EKO.

The following information as recorded per December 31, 2017 was extracted retrospectively on July 2, 2019 for each successful application from the electronic workflow and databases accessible by the DVSV:

• Austrian/European brand name
• name of marketing authorization holder (applicant)
• package sizes (as outlined in the product information sheets, e.g., in units or milliliter)
• unique tracking number (“Pharmazentralnummer”) for each package size
• ATC-Code
• defined daily dose (DDD)
• degree of innovation (1 to 8, with 8 being the highest) and degree of medical-therapeutic benefit (1 to 6, with 6 being the highest) as the outcome of the evaluation by DVSV
• date of application and date of procedural decision
• date of inclusion in the Green Box or Yellow Box, reimbursement condition (restricted or unrestricted)
• sales forecasts (number of packages) for the first 3 years in the case of a positive reimbursement decision given by the applicant.

The number of packages which were actually reimbursed over the first 3 years after inclusion into the EKO, was retrieved from the administrative database of the DVSV.

Our analysis included all applications, which 1) were handled by the DVSV between January 1, 2005 and December 31, 2014, 2) which were successful (i.e., they were listed in the EKO and thus reimbursed), and 3) which were considered to be–at least to some degree–innovative pharmaceuticals, that is the degree of innovation assigned by the DVSV was at least “me too” (i.e., with at least a degree of innovation of 5, a new substance of established pharmaceutical class). In addition, pharmaceuticals were only eligible and hence included in our study, if complete reimbursement data for three full years after inclusion into the EKO and DDD information were available. Since the EKO only covers drugs for outpatient care, we did not include hospital products, such as advanced therapy medicinal products (ATMPs).

Sales of packages of the same brand but with different sizes or strengths cannot be assumed to be independent of each other. Hence, packages of different sizes and strengths of the same pharmaceutical product (brand) with the same date of application were combined by aggregating the number of DDDs per package and regarded as a single unit/subject for the analysis. Therefore, the unit of analysis was pharmaceutical products (brands).

### Data Analysis of Forecasting Accuracy

#### Main outcome Variable

The main outcome variable was the ratio of forecasted sales to actual sales (accuracy ratio) for each product in the first, the second, and the third year after admittance into the EKO, as well as for all 3 years combined. The accuracy ratio is a relative measure of the deviation of forecasted sales from actual sales with a value of one indicating complete agreement between the number of forecasted and actual packages sold. Accuracy ratios above one indicate an overestimation while values below one indicate an underestimation of actual sales. Subtracting one from the accuracy ratio and multiplying by 100 gives the forecast error in percent. For instance, an accuracy ratio of two means that forecasted sales turned out to be twice as large as actual sales, that is, actual sales were overestimated by 100 percent. In contrast, an accuracy ratio of 0.5 means that forecasted sales were only half of actual sales, or equivalently, actual sales were underestimated by 50 percent.

A property of the accuracy ratio is that forecasts which were too low (i.e., underestimates), can deviate at maximum by 100% of actual sales. In contrast, for forecasts, which were too high, there is no upper limit. Because of this asymmetric property of accuracy ratios (Törnqvist et al., 1985; Tofallis, 2015) and the skewed nature of the data, we resorted to rank based statistics and non-parametric inferential tests throughout our analysis.

The median accuracy ratio and 95% bootstrap confidence intervals were used to quantify the typical magnitude and direction of forecasting errors. A median accuracy ratio greater or lower than one indicates a tendency to either over- or underestimate actual sales. In addition, we defined an accuracy ratio of 2, corresponding to an overestimation of actual sales by 100%, as severe overestimation and an accuracy ratio of 0.5, corresponding to an underestimation of actual sales by 50%, as severe underestimation. The proportion of forecasts which correspond to either severe over- or underestimates indicate forecasting accuracy, irrespective of the direction of forecasting errors. Further characteristics of the empirical distribution of accuracy ratios were explored graphically using box and violin plots.

The Wilcoxon rank sum test or Kruskal-Wallis test were used to evaluate group differences in the distribution of accuracy ratios between independent product groups. Dependent product groups were compared by the Friedman test for paired data. Associations between accuracy ratios and ordinal-scaled covariates were quantified by the Spearman rank correlation coefficient (with 95% bootstrap confidence intervals). Null hypotheses were rejected at a significance level *α* of 0.05 (two-sided). Because of the explorative nature of the study, we did not correct for multiple testing. All analyses were performed using R 3.6.3. (RCoreTeam, 2020).

## Results

### Description of Product Characteristics

In total, 102 pharmaceutical products (unit of analysis) were included into this study. These correspond to 211 unique packages; hence each product consists, on average, of 2.1 packages (min = 1, max = 9) of different size and/or strength. The descriptive characteristics of all 102 products included in the study are shown in Table 1.

**TABLE 1:** Descriptive characteristics of pharmaceutical products included in the study.

Product and pharmaceutical market characteristic	Categories	N (%)
Reimbursement Status	Unrestricted	21 (20.6%)
	restricted	81 (79.4%)
ATC Classification - First level	A–Alimentary tract and metabolism	17 (16.7%)
	B–Blood and blood forming organs	10 (9.8%)
	C–Cardiovascular system	14 (13.7%)
	D–Dermatologicals	2 (2.0%)
	G–Genito-urinary system and sex hormones	2 (2.0%)
	H–Systemic hormonal prep., excl. sex hormones and insulins	2 (2.0%)
	J–Antiinfectives for systemic use	16 (15.7%)
	L–Antineoplastic and immunomodulating agents	14 (13.7%)
	M–Musculo-skeletal system	3 (2.9%)
	N–Nervous system	11 (10.8%)
	P–Antiparasitic products, insecticides and repellents	0 (0%)
	R–Respiratory system	9 (8.8%)
	S–Sensory organs	1 (1.0%)
	V–Various	1 (1.0%)
Situation at the pharmaceutical market	Dynamic (A, C, J, L, or N)	72 (70.6%)
	Saturated (B, D, G, H, M, P, R, S, or V)	30 (29.4%)
Degree of innovation assessed by DVSV^a^	“me too” (“in-class” product)	62 (60.8%)
	“more innovative”	40 (39.2%)
Degree of therapeutic benefit assessed by DVSV^b^	similar benefit	65 (64,3%)
	incremental benefit	27 (26.7%)
	major benefit	9 (8.9%)

a In practice, the degree of innovation is rated on a scale from 1 (generic) to 8 (First treatment of a disease) by the DVSV (Verfahrensordnung zur Herausgabe des Erstattungskodex: Federation of Austrian Social Insurances, 2004). In this analysis we only considered products with at least a degree of innovation of 5 (i.e., “me toos”, new compound of established pharmaceutical class) or higher. For simplicity, we grouped products with a degree of innovation of 6, 7, or 8 into the “more innovative” category.

b In practice, the degree of therapeutic benefit is rated on a scale from 1 (No added therapeutic value, generic) to 6 (Substantial added therapeutic benefit for the majority of patients) by the DVSV(17). For simplicity, we grouped products with a degree of therapeutic benefit of 3 or 4 into the “incremental benefit” category and 5 or 6 into the “major benefit” category. The degree of therapeutic benefit was not available for one product because it was not rated by the DVSV.

### General Accuracy of Pharmaceutical Sales Forecasting

**FIGURE 1:**
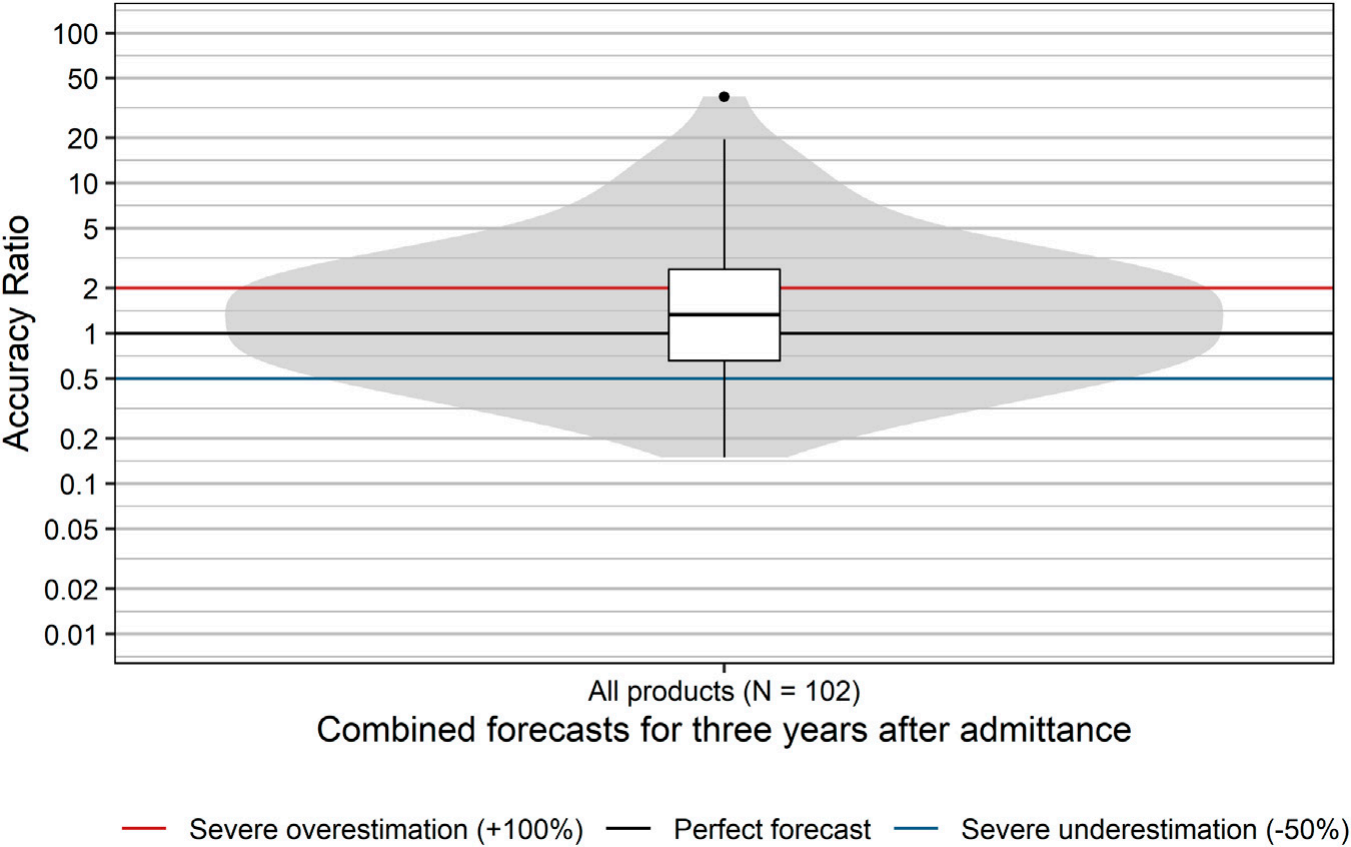
Distribution of accuracy ratios of products with a positive reimbursement decision. Box plots and violin plots visualize the distribution of accuracy ratios of combined forecasts for the 3 years after admittance. Due to their asymmetric nature, accuracy ratios are plotted on a logarithmic (base 10) scale to avoid visually overemphasizing overestimates compared to underestimates.

The accuracy of sales forecasts for the first 3 years after admittance into the EKO showed high variability with accuracy ratios ranging from 0.15 (i.e., underestimation by 85%) to 37.5 (i.e., overestimation by 3,650%; Figure 1). Sales forecasts of applicants were significantly more often too optimistic, with forecasts for nearly two thirds of products (60.8%) overestimating actual sales (*p* = 0.037; binomial test). The median accuracy ratio [95% CI] was 1.33 [1.03; 1.74] corresponding to an overestimation of 33%. Moreover, for 37.3% of all examined products, forecasts severely overestimated actual sales by more than 100%. Severe underestimation by more than 50% was seen for 18.6% of all products. Thus, forecasts of actual sales for 55.9% of all examined products severely over- or underestimated actual sales for the 3 years after admittance into the EKO.

**FIGURE 2:**
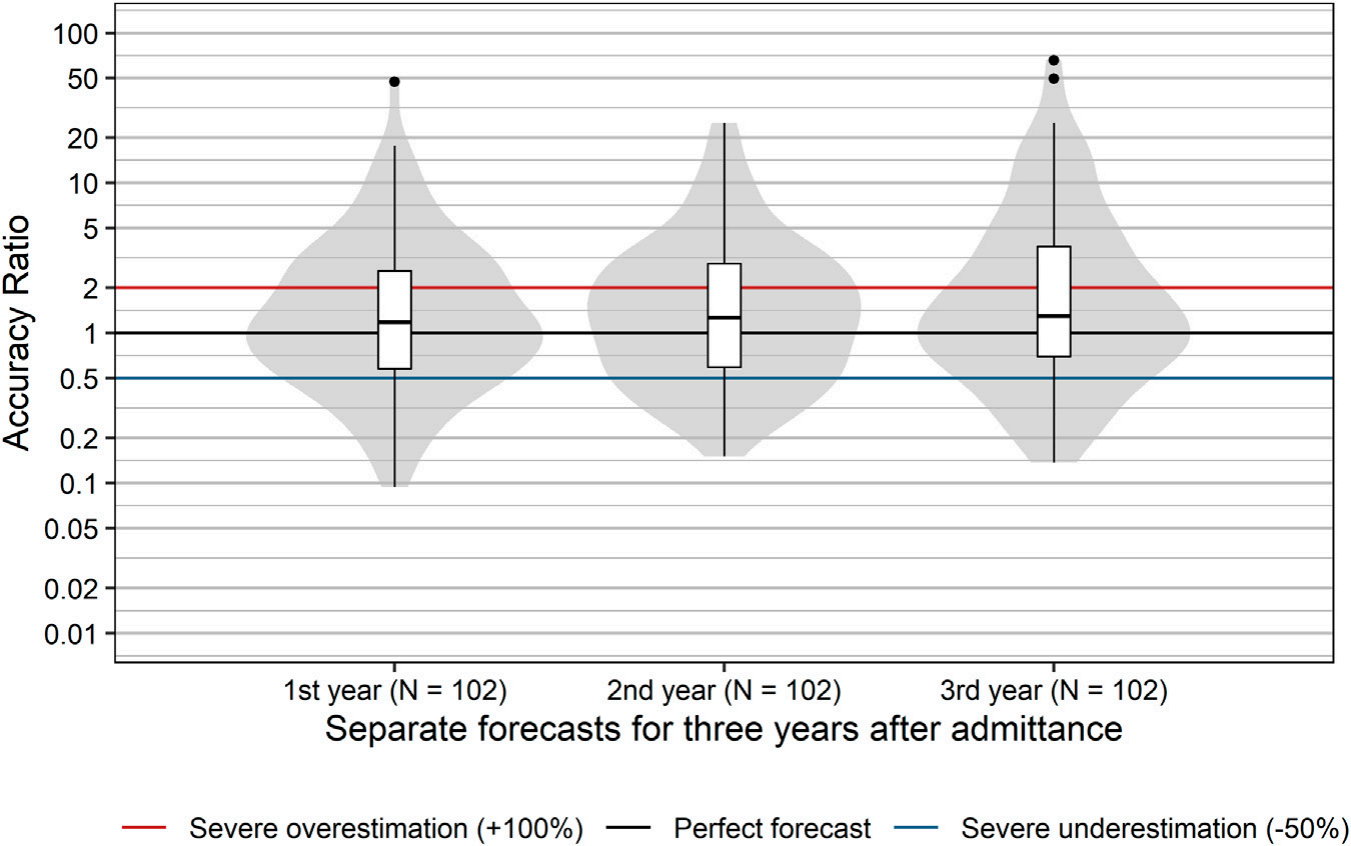
Distribution of accuracy ratios of products with a positive reimbursement decision. Box plots and violin plots visualize the distribution of accuracy ratios separately for the first, second, and third year after admittance. Due to their asymmetric nature, accuracy ratios are plotted on a logarithmic (base 10) scale to avoid visually overemphasizing overestimates compared to underestimates.

The distribution of accuracy ratios differed significantly between the first, second, and third year after admittance to the EKO (*p* = 0.006; Friedman test), with median accuracy ratios [95% CI] increasing from 1.18 [0.92; 1.52] in the first year to 1.30 [1.00; 1.72] and 1.34 [1.00; 1.83] in the second and third year, respectively, (Figure 2). Descriptively, the proportion of forecasts severely over- or underestimating actual sales increased slightly from 50% in the first year to 55% in the second year and 56% in the third year after inclusion in the reimbursement list. In summary, the majority of sales forecasts showed rather severe inaccuracy, tended to be too optimistic, and this was particularly true for later years after admittance.

### Potential Prognostic Factors of Sales Forecast Accuracy

Several factors could conceivably account for the poor accuracy of sales forecasts (Park et al., 2016). These may concern the disease category, the market situation of the product, conditional vs. unconditional reimbursement, the degree of innovation or the degree of added therapeutic benefit, as compared to existing standards. Accordingly, we systematically explored the relation of each individual variable to the accuracy ratio.

### Disease Area (ATC- Code Level 1)

**FIGURE 3:**
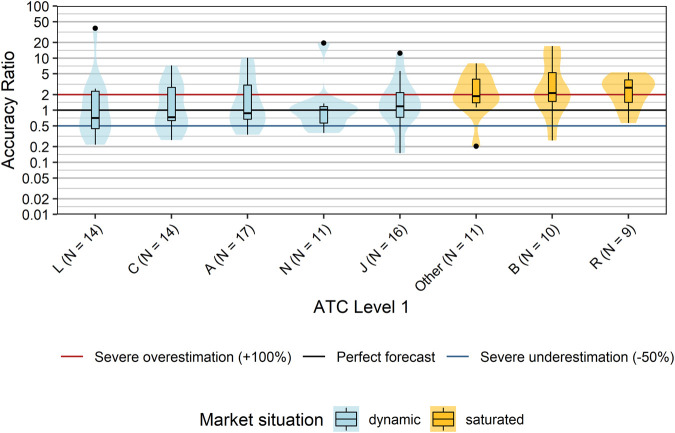
Comparison accuracy ratios grouped by disease areas (ATC Classification Level 1). Box plots and violin plots show accuracy ratios of forecasted sales in the 3 years after a positive reimbursement decision grouped by disease category. For each disease category, the market situation of the majority of products within this disease category is indicated by different colors. ATC-L1 groups D, G, H, M, S, and V each contained only three or less products, and were combined into one “Other” category. Due to their asymmetric nature, accuracy ratios are plotted on a logarithmic (base 10) scale to avoid visually overemphasizing overestimates compared to underestimates. Abbreviations: ATC, Anatomical Therapeutic Chemical; D, Dermatologicals; H, Systemic hormonal preparations, excluding sex hormones and insulins; M, Musculo-skeletal system; S, Sensory organs.

We first grouped products according to disease areas using their ATC-codes at level 1 (ATC-L1). With the exception of ATC-L1 P (antiparasitic products, insecticides and repellents), at least one innovative product was admitted into the EKO for each possible ATC-level 1. However, the number of products was highly variable ranging from one product with ATC-L1 S (Sensory organs) to 17 with ATC-L1 A (alimentary tract and metabolism). In order to guarantee anonymity of products we combined all ATC-L1 with three or less products (D, G, H, M, S, V) into one “Other” category. We did not find a statistically significant difference in the distribution of accuracy ratios based on disease areas/ATC level 1 (*p* = 0.15, Kruskal-Wallis Test; Figure 3). Descriptively, the highest median accuracy ratios and–hence most pronounced overestimations–were observed for products with ATC-L1 R (respiratory system; median accuracy ratio of 2.69) and ATC-L1 B (Blood and blood forming organs; median accuracy ratio of 2.14). A median accuracy ratio <1 indicating an underestimation of typical sales forecasts was seen for products with ATC-L1 L (antineoplastic and immunomodulating agents; median accuracy ratio of 0.71), ATC-L1 C (cardiovascular system, median accuracy ratio of 0.74), and ATC-L1 A (Alimentary tract and metabolism; median accuracy ratio of 0.87).

### Situation at the Pharmaceutical Market

**FIGURE 4:**
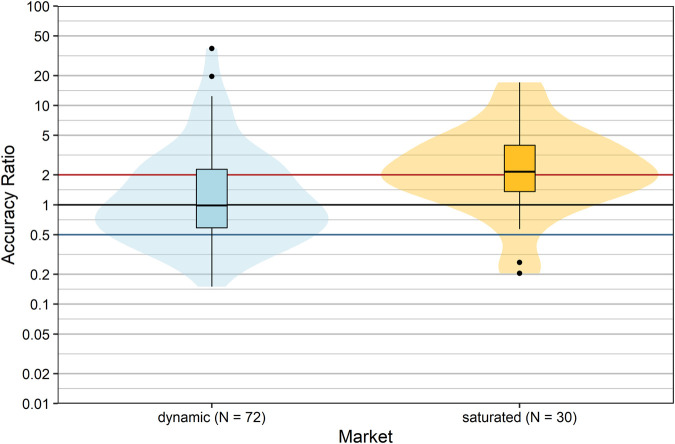
Comparison of accuracy ratios by market dynamics. Box plots and violin plots show accuracy ratios of forecasted sales in the 3 years after a positive reimbursement decision grouped according to market situation. Due to their asymmetric nature, accuracy ratios are plotted on a logarithmic (base 10) scale to avoid visually overemphasizing overestimates compared to underestimates.

Snider et al. suggested that the state at a defined market can greatly influence the accuracy of budget impact estimation (Snider et al., 2019). We hypothesized that if a product is intended for use in a therapeutic area in which the R&D (research and development) rate is high (“dynamic market”), it is difficult to estimate future sales, because the market entry of competitive drugs cannot be anticipated. Therefore, we allocated products to two market situations (“dynamic” vs. “saturated”) according to their ATC classification, taking into account the number of pipeline drugs including potential first-in-class medicines per therapeutic area/disease (dynamic: A, C, J, L, or N; saturated: B, D, G, H, M, P, R, S, or V). The forecast accuracy of products allocated to a dynamic market significantly differed from products allocated to saturated markets (*p* = 0.002, Wilcoxon rank sum test; Figure 4). In saturated markets, companies tended to severely overestimate actual sales with a median accuracy ratio [95%CI] of 2.15 [1.63; 3.50], i.e. by 115%. In contrast, forecasts of products in dynamic markets showed a median accuracy ratio [95% CI] of 0.98 [0.72; 1.33] with a slight underestimation of actual sales by 2%. However, the proportion of forecasts severely over- or underestimating actual sales was 52.8% for products in dynamic markets compared to 63.3% for products in saturated markets, and hence forecast accuracy was low for the majority of products irrespective of market dynamics.

### Reimbursement Status

**FIGURE 5:**
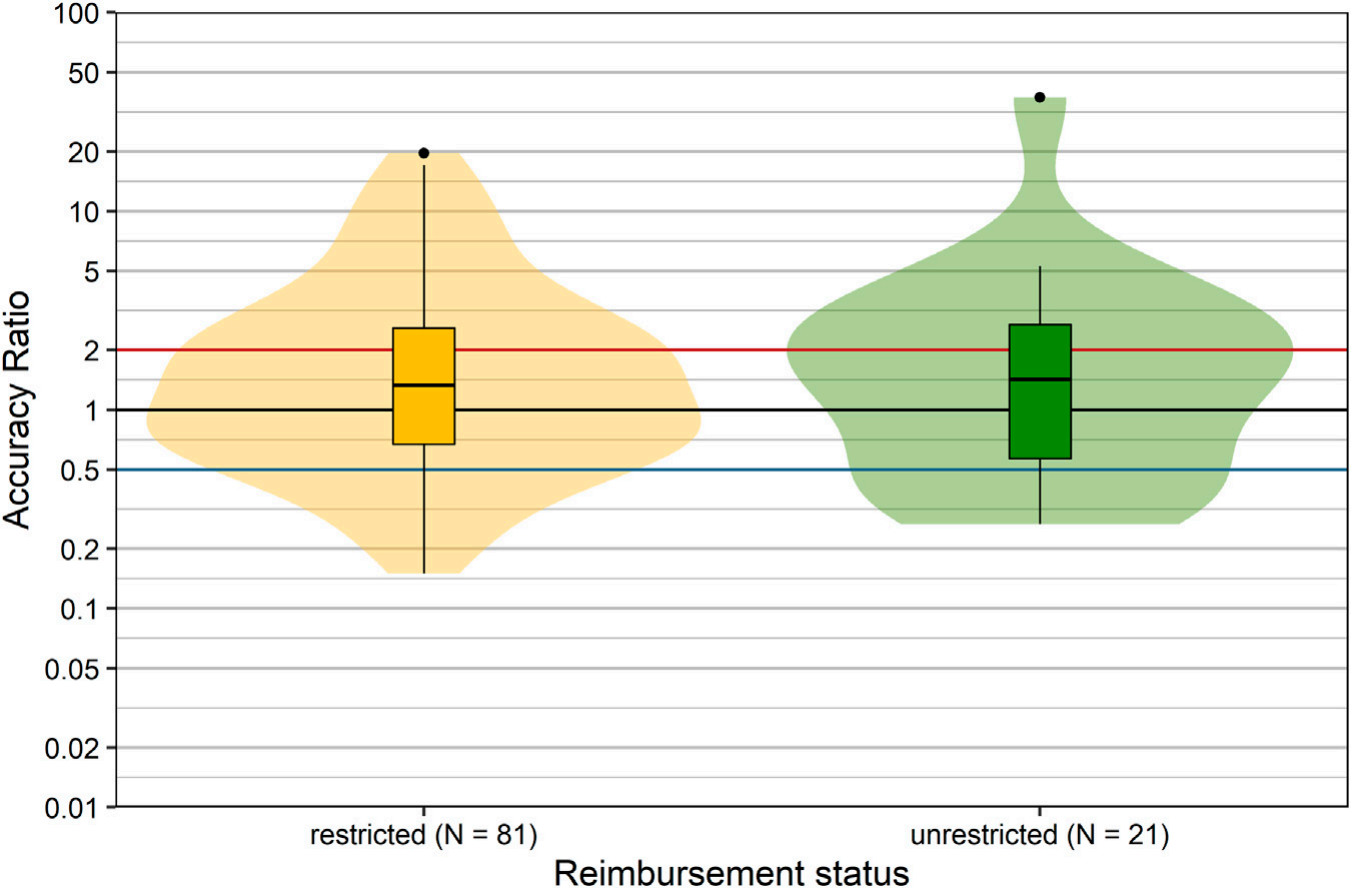
Comparison of accuracy ratios by reimbursement status. Box plots and violin plots show accuracy ratios of forecasted sales in the 3 years after a positive reimbursement decision, grouped according to reimbursement decision. Due to their asymmetric nature, accuracy ratios are plotted on a logarithmic (base 10) scale to avoid visually overemphasizing overestimates compared to underestimates.

In Austria, products are reimbursed with or without restrictions. The reimbursement status may impact the utilization of a product in real-life situations. Therefore we compared the forecast accuracy of products with different reimbursement status (restricted vs. unrestricted): it is apparent from inspection of Figure 5 that the distributions of accuracy ratios were nearly identical (*p* = 0.934, Wilcoxon rank sum test). Thus, the forecast accuracy showed no association with the reimbursement status.

### Degree of Innovation and Therapeutic Benefit

Innovative pharmaceuticals have the potential to provide therapeutic benefits for patients and to generate high profits for manufacturers. Usually, the level of uncertainty with respect to the magnitude of the therapeutic effect and the uptake is higher than for established products. Thus, the prediction of sales is expected to be more difficult for more innovative products, due to lack of precedents. We explored this conjecture by comparing the forecast accuracy with respect to the degree of pharmacological innovation (as assessed by the DVSV), grouped into me-too/less innovative drugs and more innovative drugs.

**FIGURE 6:**
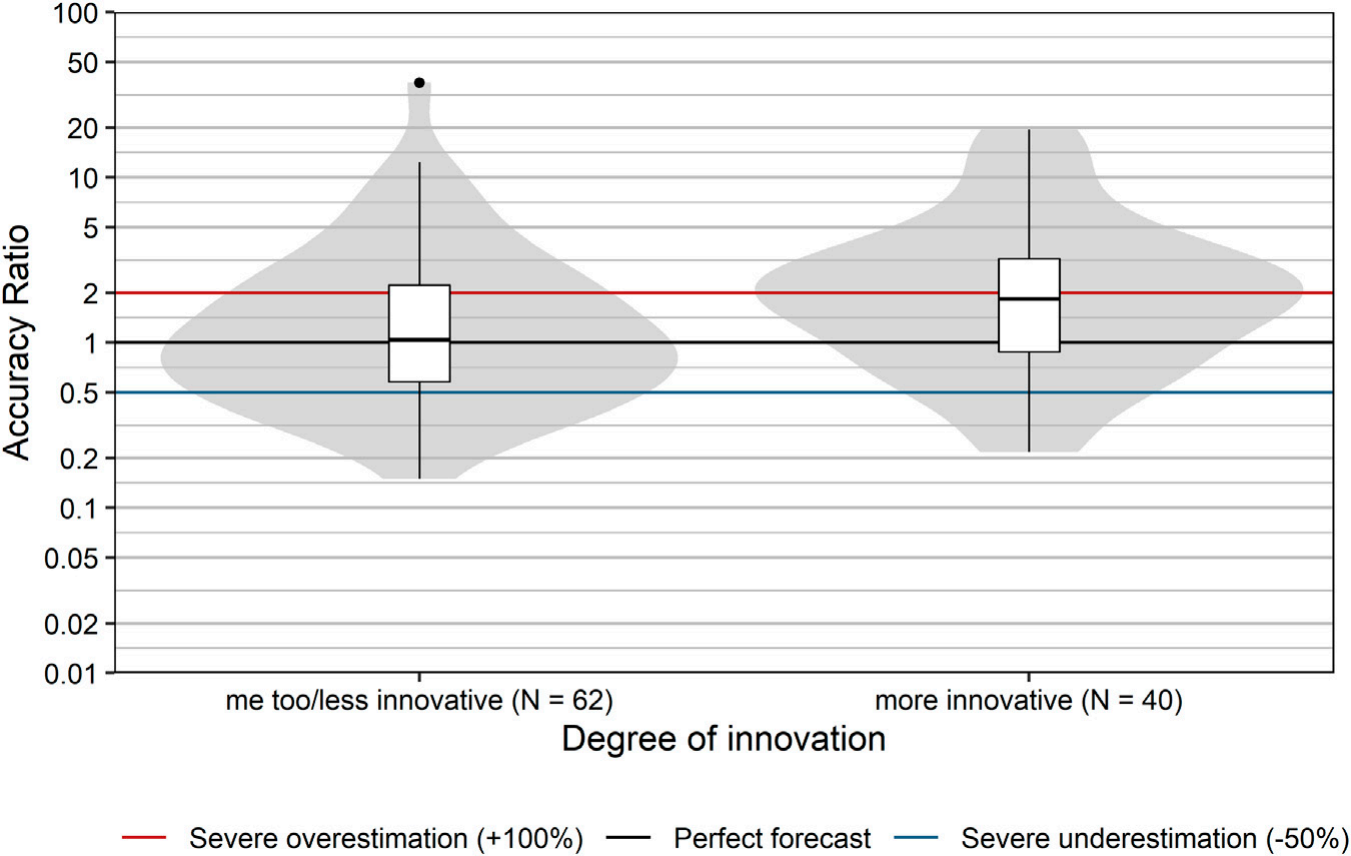
Comparison of accuracy ratios by degree of innovation. Box plots and violin plots show accuracy ratios of forecasted sales in the 3 years after a positive reimbursement decision, grouped by degree of innovation as assessed by DVSV. Due to their asymmetric nature, accuracy ratios are plotted on a logarithmic (base 10) scale to avoid visually overemphasizing overestimates compared to underestimates.

Accuracy ratios significantly differed between more innovative products and me-too/less innovative products (*p* = 0.025, Wilcoxon rank sum test; Figure 6). Sales of more innovative products tended to be overestimated with a median accuracy ratio [95% CI] of 1.84 [1.26; 2.51], while me-too/less innovative products showed only a minor typical overestimation with a median accuracy ratio [95% CI] of 1.04 [0.74; 1.35]. Severe over- or underestimation was observed for 60 and 53.2% of more innovative and me-too/less innovative products, respectively.

**FIGURE 7:**
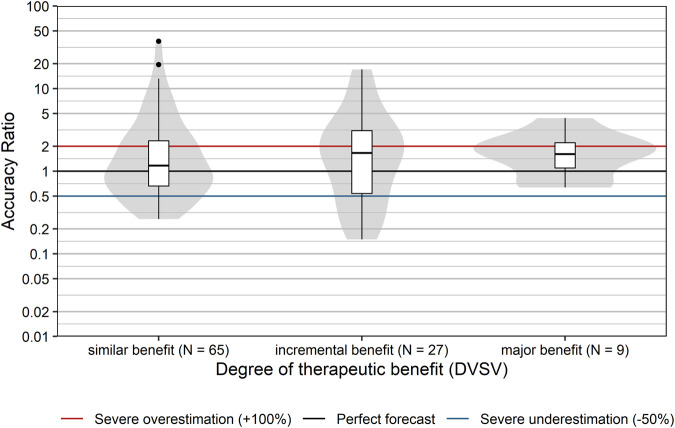
Comparison of accuracy ratios by degree of therapeutic benefit. Box plots and violin plots show accuracy ratios of forecasted sales in the 3 years after a positive reimbursement decision grouped by degree of therapeutic benefit as assessed by DVSV. Due to their asymmetric nature, accuracy ratios are plotted on a logarithmic (base 10) scale to avoid visually overemphasizing overestimates compared to underestimates.

Finally, we also examined whether the classification based on the degree of therapeutic benefit (as assessed by the DVSV) affected sales forecast accuracy. Presumably, the knowledge base is larger for products which provide a similar or comparable therapeutic benefit to already available reimbursed products on the market, than for those providing an incremental benefit, or major benefit. However, our data do not confirm this assumption (Figure 7). The degree of therapeutic benefit was not significantly correlated with forecast accuracy ratios (r_s_ [95% CI] = 0.06 [−0.13; 0.25], *p* = 0.538). This was also true for the correlation between the degree of therapeutic benefit and severe over- or underestimation (r_s_ [95% CI] = −0.03 [−0.22; 0.17], *p* = 0.785).

### Accuracy of Pharmaceutical Forecasting Over Time

**FIGURE 8:**
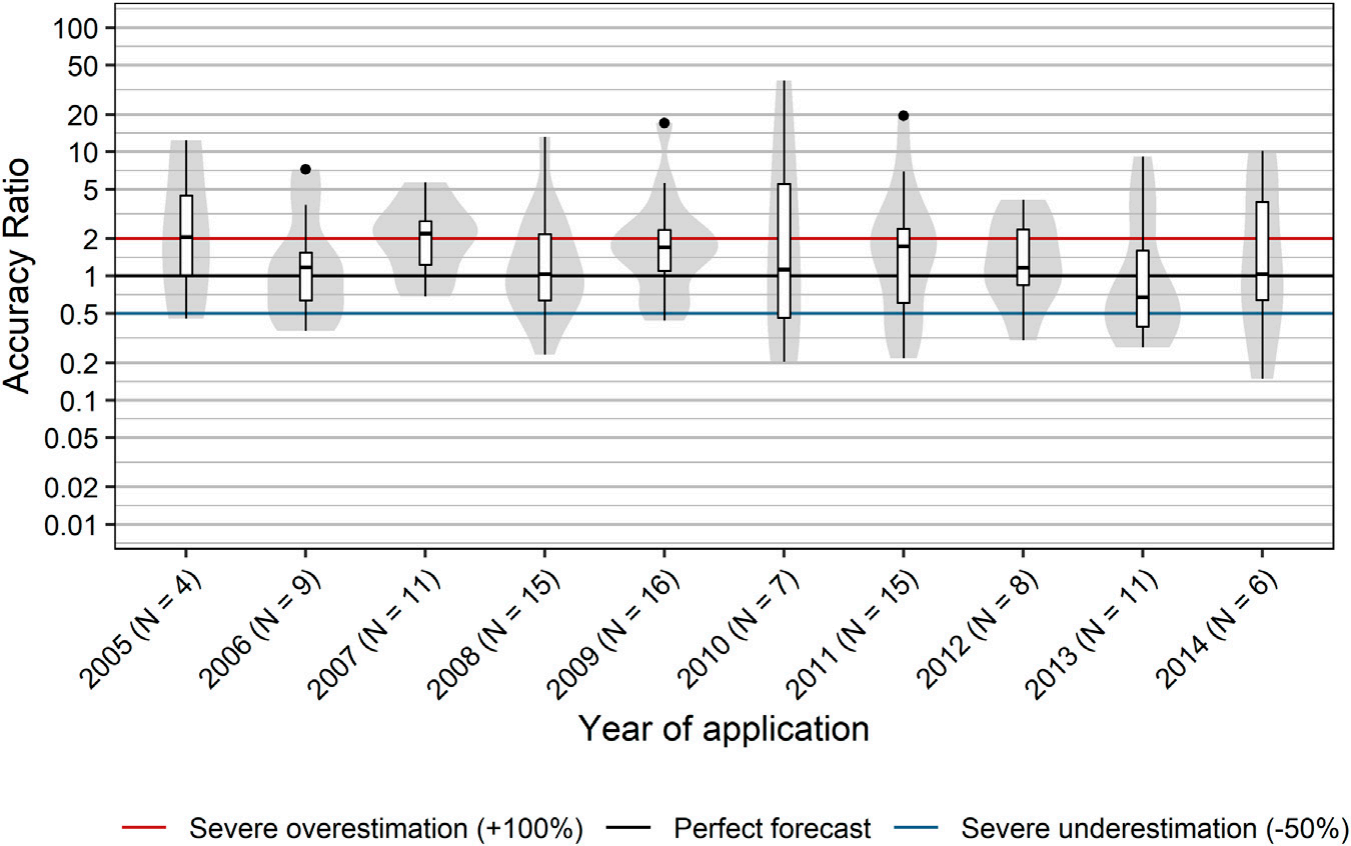
Accuracy of sales forecasts over time. Box plots and violin plots show accuracy ratios of forecasted sales in the 3 years after a positive reimbursement decision, grouped by year of application. Due to their asymmetric nature, accuracy ratios are plotted on a logarithmic (base 10) scale to avoid visually overemphasizing overestimates compared to underestimates.

It is reasonable to assume that sales forecasts may improve over time, because companies are expected to learn from sales data from the past. However, we did not find a systematic time-dependent trend in accuracy ratios from 2005 to 2014 (Figure 8). No significant correlation between year of application and accuracy ratios was observed (r_s_ [95% CI] = −0.10 [−0.30; 0.11], *p* = 0.300). In addition, the year of application did not show an association with severe over- or underestimates of actuals sales (r_s_ [95% CI] = 0.01 [−0.18; 0.20], *p* = 0.914). Hence, our data do not suggest a relevant learning effect on the aggregate level.

## Discussion

Competent authorities and/or payers have adopted different approaches to the pricing and reimbursement of new medicines (Godman et al., 2018). If budget impact forecasts are to be informative for reimbursement evaluations, their accuracy needs to be known. Although sales forecasts for new drugs are widely available, a follow-up of their accuracy is much harder to come by. Our study systematically examines the accuracy of sales forecasts for innovative drugs over a decade for the outpatient sector in a national market. We found that the majority of sales forecasts provided by applicants were highly inaccurate. In most instances, forecasts were too optimistic, in particular for later years after admittance. Our analysis revealed several surprising findings:

(i) Forecasting of sales was — on average — more accurate for drugs entering a dynamic pharmaceutical market than for products which compete with existing products in a saturated market. This observation is counterintuitive. The expectation is that sales could be more readily predicted if a drug enters a more mature market, where the number of eligible patients and the sales of the existing products are known. Consistent with this expectation, sales forecasts were less divergent for me-too/less innovative drugs than for innovative compounds. It is thus not clear why companies seemed to be better at forecasting their sales in dynamic markets.
(ii) The anticipated therapeutic value of a new product also showed no association with forecast accuracy. However, we note that only nine of all 102 products were assigned a major benefit over existing treatment options. This number may have been too low to reveal an underlying association between an anticipated therapeutic value and forecasting accuracy. This may be related to the allocation of resources (see below).
(iii) We posited that forecasting accuracy would improve over time. In fact, Cha et al. found that consensus forecasts improved over time as more information became available (Cha et al., 2013). However, our data do not suggest a relevant learning effect on the aggregate level during the analyzed period from 2005 to 2014. This is again surprising, because companies are expected to gain experience from earlier reimbursement procedures, and past trends are expected to improve future sales forecasting.

Our findings are in line with a recent analysis, which covered a more limited number of products (i.e., 49) and a similar time interval (between May 2005 and December 2013) in the Welsh branch of National Health Service/NHS (Keeping et al., 2019): actual expenditures indicated that companies overestimated their sales in the vast majority of their submissions (i.e., 82%). In addition, the sales/expenditures fell progressively short of expectations (i.e., on average by 41% in the first year and 62% in the third year). Our analysis is based on a larger data set in the Austrian context, but is consistent with several findings, i.e., a median accuracy ratio clearly above one and a progressive deviation of actual from projected sales over the first 3 years.

Initial overestimates may be accounted for by clinical inertia on the side of prescribers, possibly abetted by signals for cautious prescribing of new, expensive medicines from payers wishing to manage the entry of new medicines (Godman, 2021). In subsequent years, the discrepancy between forecasts and actual uptake may arise–at least in part–from other factors, which are not adequately considered in the projections. For instance, a lower degree of therapy adherence in “real life” vs clinical trials, or the fact that “hyped” drugs turn out not to fully measure up to the high initial clinical and market expectations.

Forecasting sales of a new product already at the time of applying for reimbursement is further complicated by not knowing the exact outcome of the application. In Austria, initial sales forecasts are not revised during the assessment of the submission and subsequent negotiations. This means actual reimbursement conditions can differ from the initial application and if so, are typically more restrictive. Plausibly, changing reimbursement conditions after submission would negatively influence the accuracy of the original forecasts. However, we did not observe clear differences between a restricted vs unrestricted reimbursement status and forecast accuracy.

Evidently, the low accuracy of pharmaceutical sales forecasts and resulting budget impact estimates are a widespread problem. Also, this applies not only to pharmaceutical sales forecasts submitted by market authorisation holders to payers. In a budget impact analysis from Brazil, the estimated costs for adalimumab for the treatment of rheumatoid arthritis were overestimated by 463% (US $ 4.7 billion) due to uncertainties in the number of patients treated and to variations in prices (Faleiros et al., 2021). In a study from Thailand, the theoretically estimated constant budget impact per patient and year for the treatment of lung cancer with pemetrexed was initially overestimated by 34% (US $ 11,881 vs. 8,834) (Sooksriwong and Chanjaruporn, 2011). However, actual expenditures per patient and year increased over time to US $ 10,053 and hence came closer to the initial constant budget impact estimate, which highlights the importance of time trends for the accuracy of budget impact analyses. For 16 new drugs launched in the United States between 2012 and 2016, Broder et al. (Broder et al., 2018) identified 25 sales forecasts from diverse sources, i.e. consulting companies, analyst companies and a non-profit organization (ICER, Institute for Clinical and Economic Review). These estimates differed widely from actual sales (accuracy ratio 0.2–37.5), but on average the estimate was 5.5-fold higher than the actual sales. We note that these overestimates are considerably larger than those which we observed.

Overestimation may be due to optimism on the side of analysts and of the marketing authorisation holder. In Austria, the Code of Reimbursement (EKO) specifies that sales forecasts and budget impact calculations submitted by companies should be based on empirical evidence and available data, such as the prevalence of the disease to be treated. However, it is not transparent how the calculations are actually carried out. The quality and amount of resources invested in the forecasts is likely to affect their accuracy. There is circumstantial evidence to support this conjecture: forecasts by large companies were noted to be more accurate than those provided by smaller enterprises (Cha et al., 2013). Large companies with publicly traded shares must take pains that their submitted forecasts are not in conflict with forecasts published for investors. These forecasts are likely to err on the optimistic side.

Another possible explanation of the observed tendency to overestimate actual sales may be that expected sales are reached and finally even surpassed, but with a temporal delay, as market penetration may need more time than originally assumed at the time of the application for reimbursement. In addition, the tendency to overestimate actual sales was primarily observed for products in saturated markets. For products in several disease areas classified as dynamic markets (e.g., ATC-Level 1 L), even a clear tendency to underestimate actual sales was observed. Although these differences between single disease areas did not reach statistical significance in our study, this could be explained by a lack of statistical power due to the relatively large number of compared disease groups and given the moderate sample size. Hence, these explorative findings might still be worthwhile to examine in future research.

On the other hand, payers are also not immune to forecasting inaccuracies. This is exemplified by the projected budget impact estimates, which the Dutch National Healthcare Institute (ZIN) calculated for directly acting antivirals in the treatment of hepatitis C (Geenen et al., 2019): these forecasts (€388 to 510 million) were made in compliance with the most recent ISPOR guideline (Sullivan et al., 2014), but they nevertheless overestimated the actual budget impact (€ 248 million). The range of overestimation (by 41–105%) was in line with our results. Overestimation of future sales raises concerns with respect to affordability, in particular if large patient populations are involved. This may lead to initial restrictions on the use of new medicines, as was widely the case with the novel direct-acting antivirals against hepatitis C (Barua et al., 2015).

In the case of orphan drugs, small patient populations are concerned, but high prices may lead to impactful budget estimates, which, in turn, can affect the accessibility of treatment for patients. This is the case in Central and Eastern Europe (Malinowski et al., 2019). There are several examples where high budget impact estimates were associated with restricted reimbursement to a specific patient population under defined conditions (Niezen et al., 2006; Lo Re et al., 2016; American Society of Clinical Oncology, 2018; Geenen et al., 2019; Malinowski et al., 2019). Overestimating the budget impact may, therefore, negatively affect access to medicines.

It is of no less concern that sales performance of a remarkable number of products was highly underestimated. Payers are responsible for sustainability of healthcare systems and have to ensure prudent use of limited resources. For payers, underestimation can be especially troubling when considering alternative pricing models such as those suggested for cancer medicines (Niezen et al., 2006; Niezen et al., 2009; American Society of Clinical Oncology, 2018). Underestimation may lead to choosing a scheme that does not adequately account for an unexpected increase in use and may later require re-negotiating the price with a reluctant company. It may be speculated that some budget estimates are based less on models than strategic marketing considerations—that is, companies trying to “game” reimbursement systems (Pekarsky, 2015).

Taken together, our data confirm that there are significant challenges with forecasting sales of newly reimbursed pharmaceuticals and hence their budget impact. In the light of these findings, we do not consider these estimates as reliable for a prudent decision-making process. Several studies have pointed out the low methodological standards and weaknesses of published budget impact analyses (van de Vooren et al., 2014; Mauskopf and Earnshaw, 2016). However, our data did not allow evaluating the quality of methodological standards used by companies to estimated future sales. Hence, it remains unclear whether low accuracy of submitted forecasts is based on potentially subpar methodological forecasting approaches, unexpected changes in patient needs and market dynamics, or strategic considerations of companies when applying for reimbursement. We suggest that cross-system and multi-national comparisons ought to be helpful for identifying further factors which might affect forecasting accuracy. This requires standardization of methodology, e.g., based on the ISPOR report (Sooksriwong and Chanjaruporn, 2011). We are aware of the fact that this per se cannot solve the problem (Faleiros et al., 2021). However, valid international, cross-system comparisons may be useful to provide answers to the following non-exhaustive list of questions: are prevalence data consistently inaccurate? How reliable is the derivation of the target population from the prevalence data? Which data are needed to improve estimates of uptake? Are these data missing or underutilized?

## Conclusion

We show that the majority of pharmaceutical sales forecasts provided by applicants for reimbursement in Austria were highly inaccurate and were on average too optimistic. The accuracy of sales forecasts ought to be improved, if they are to be useful for reimbursement procedures.

## Data Availability

Forecasted sales examined in this study were submitted by pharmaceutical companies as part of the reimbursement appraisal procedure in Austria. As this information is confidential by Austrian law (see Rule of Procedure for Publishing the code of Reimbursement, Verfahrensordnung zur Herausgabe des Erstattungskodex, and Article 20 of the Austrian Constitution), the raw data can not be made publicly available. The same applies to the data regarding the number of reimbursed packages; this information is protected by § 26a ff Federal Competition Act. R code underlying all results within this work is available at https://osf.io/upy97/.

## Abbreviations

ATC, Anatomical Therapeutic Chemical; DVSV, Dachverband der österreichischen Sozialversicherungen [Austrian Federation of Social Insurances]; BI, budget impact
